# An RBF Meshless Approach to Evaluate Strain Due to Large Displacements in Flexible Printed Circuit Boards

**DOI:** 10.3390/mi13081163

**Published:** 2022-07-22

**Authors:** Corrado Groth, Andrea Chiappa, Stefano Porziani, Pietro Salvini, Marco Evangelos Biancolini

**Affiliations:** 1 Enterprise Engineering Department, Università degli Studi di Roma “Tor Vergata”, Via del Politecnico 1, 00133 Roma, Italy; corrado.groth@uniroma2.it (C.G.); salvini@uniroma2.it (P.S.); biancolini@ing.uniroma2.it (M.E.B.); 2 RBF Morph srl, Via Rosmini 4, 00077 Montecompatri, Italy; andrea.chiappa@rbf-morph.com

**Keywords:** radial basis functions, meshless, large displacements, flexible printed circuit boards

## Abstract

Thin plates are very often employed in a context of large displacements and rotations, for example, whenever the extreme flexibility of a body can replace the use of complicated kinematic pairs. This is the case of the flexible Printed Circuit Boards (PCBs) used, for example, within last-generation foldable laptops and consumer electronics products. In these applications, the range of motion is generally known in advance, and a simple strategy of stress assessment leaving out nonlinear numerical calculations appears feasible other than desirable. In this paper, Radial Basis Functions (RBFs) are used to represent a generic transformation of a bi-dimensional plate, with all the derivate fields being analytically achieved without the need for a numerical grid for large-displacement applications. Strains due to bending are easily retrieved with this method and satisfactorily compared to analytical and shell-based Finite Element Method (FEM) benchmarks. On the other hand, the computational costs of the juxtaposed methods appear far different; with the machine being equal, the orders of magnitude of the time elapsed in computation are seconds for the RBF-based strategy versus minutes for the FEM approach.

## 1. Introduction

**Figure 1:**
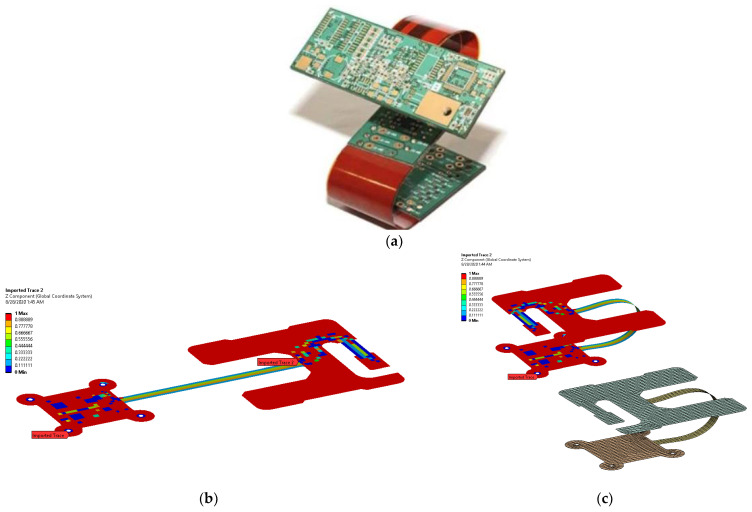
Example of flexible-PCB study [15] (**a**) real flexible PCB in mounting configuration, (**b**) FEM model in un uninstalled state with trace mapping and (**c**) FEM model in installed state with and without trace mapping.

At present, the Finite Element Method (FEM) is the established standard for stress assessment, especially when complex geometries are under study [1]. The discretization of the domain into smaller elements, where linear or quadratic basis functions approximate the local displacement field [2], allows complicated shapes and boundary conditions to be handled. On the other hand, the generation of the numerical grid is often an expensive task [3], able to negatively affect the results if not properly carried out. This aspect is further exacerbated if the transformation of the body is such to degenerate element shapes, as in the case of large deformations [4] or moving discontinuities [5], with the consequent need to update the mesh during the analysis. Meshless methods [6] supply an alternative approach to the FEM for the study of continuum mechanics and physics problems in general. The computational domain is covered with a series of points, both within and at the boundary, that provide the basis to construct an approximate solution. Characteristics such as the continuity and smoothness of the results as well as good scalability to higher dimensions make mesh-free methods appealing choices for practical use in many applications. Lucy [7] was the first to study a complex astronomical phenomenon without a mesh, only considering a set of interacting points. Later on, Libersky et al. [8] employed the same method for elasto-plastic solids, dealing with large distortions. The methods of diffused approximation (DA) [9] and the partition of unity [10] decompose the computational space into smaller patches, partially superimposed in order to guarantee the continuity of the representation. A drawback of meshless methods is that, when integration over the domain is necessary, a grid is introduced in any case, even if its construction could be someway released from the particular geometry, as showed by Belytscho and co-workers [11]. The local Petrov–Galerkin method [12] prescribes the subdivision of the global domain into regular overlapping subdomains, in which integration evaluates in a truly meshless fashion relying on local weak forms. In this paper, a simple strategy to study the large deflections of a thin plate is proposed, circumventing all the shortcomings of an FEM-based approach, e.g., the preparation and handling of a non-linear analysis. A generic transformation, including both displacement and deformation, is described on a point-wise basis relying on Radial Basis Functions (RBFs) that supply all the derived fields via analytical, and thus exact, differentiation. Such an approach is well-suited whenever the kinematics of the component are already defined at the design stage, since a prescribed range of motion needs to be covered. This is the case of the flexible printed circuit boards (PCBs) that must be compliant with the folding pattern of the electronic devices where they are installed. The numerical analysis of PCBs in the usual sense is challenging [13,14]; due to the particular installed configuration of flexible PCBs (see Figure 1a), high modeling efforts are required to properly catch large-displacement effects, and specific numerical modeling techniques, such as “trace mapping”, have to be applied (see Figure 1b,c). Moreover, even when the range of motion is strictly guided and known in advance, the computational difficulties associated with the nonlinear contacts arise. Given these challenging requirements needed for the analysis of strains in deformed PCB components, the idea to study this problem with a tailored procedure, taking full advantage of all the peculiarities of the specific case, gains high interest. While a full non-linear finite-element analysis (FEA) of such structures into complex shapes is indeed possible, it is also expected to be computationally expensive (even with high-performance computers). A recent research study in this field by the authors shows how RBFs can be effectively used to morph such trace-mapped flexible PCBs into complex shapes [15].

The procedure here exposed builds on this and is intended as a first step in the direction of calculating stresses and strains on flexible PCBs subject to a prescribed bending, exploring the capability of a meshless method in retrieving strains in a thin structure undergoing large displacements.

The proposed approach allows such strains to be calculated whenever the target shape is known in advance and in pure bending, for example, when the motion is guided by a known folding kinematic. In this case, strains on the PCB can be computed in a meshless fashion and without recurring to an FEM simulation by directly defining its original and final geometries. The paper is arranged as follows: Section 1 introduces the problem and presents the state-of-art techniques available to tackle it. After recalling a basic mathematical background on RBFs, in Section 2, the mathematical foundation of the proposed procedure is exposed, approaching the flexible PCB as a 2D object and supplying a simple but comprehensive method to describe the kinematics of a generic transformation field by means of an RBF interpolant. In Section 3, the method is first demonstrated on two analytical transformations for which an exact result can be easily computed, and strains are compared to those obtained with FEM computation; then, a more complex problem, representative of an application relevant for the flexible-PCB industry, is faced using both the proposed approach and a nonlinear FEM simulation with contacts. In Section 4, the results are presented and discussed to close the article.

## 2. Mathematical Background

### 2.1. Radial Basis Functions

Radial Basis Functions were introduced during the 1960s to deal with problems of multidimensional interpolation [16]. During the successive decades, their great versatility allowed applications in many fields to be achieved, even far from the original scope, such as neural networks [17], surface reconstruction in computer graphics [18], mesh morphing [19,20] and image analyses of deformations [21], to cite a few. RBF mesh morphing is employed for several applications, from FSI coupling [22] to genetics [23] and from evolutionary optimizations [24] to advanced modeling [25]. The meshless nature of RBFs makes them natural candidates whenever a continuous representation should be constructed upon granular data [26] or when information needs to be released from mesh specificity [27,28]. Of course, RBFs can also be the basic ingredients of truly mesh-free methods [29,30], where differential equations are solved without the frame of a numerical grid [31].

The main idea behind RBF interpolation is that information at any location x of the space can be composed bottom-up, from the contributions of N source points (with coordinates $x_{i}$), where data are given. Each source point participates with a radial basis φ—a function of its Euclidean distance from target point x—weighted by a coefficient $\gamma_{i}$. RBF interpolator $s\left(x\right)$ collects all the contributions from the source points:

(1) $$s\left(x\right)=\overset{N}{\underset{i=1}{\sum }}\gamma_{i}\varphi \left(\left\|x-x_{i}\right\|\right)$$

In Equation (1), biases $\gamma_{i}$ are unknown; they are derived from imposing the exact retrieval of the given data, $g_{i}$, at the source points. In matrix notation, if γ and g are the vectors containing $\gamma_{i}$ and $g_{i}$, respectively, and M is the matrix that collects, row-by-row, the radial bases calculated at each source point, the retrieval condition allows one to determine vector γ of coefficients:

(2) Mγ=g

In many cases, a polynomial term $\;h\left(x\right)$ is added to the series in Equation (1); in this way, the RBF interpolant can exactly reproduce those functions of the same kind of $h\left(x\right)$ also between the source points. An orthogonality condition is introduced in the system of Equation (2) and modifies it accordingly [32], in order to determine the coefficients within the polynomial.

RBF interpolation as described so far is able to assign a scalar value to each point of the interpolation space. Nonetheless, a vector field can also be reproduced as long as a different RBF interpolant represents each component, e.g., in a 3D space:

(3) $$\left\{\begin{array}{c} s_{x}\left(x\right)=\overset{N}{\underset{i=1}{\sum }}\gamma_{i}^{x}\varphi \left(\left\|x-x_{i}\right\|\right)+h_{x}\left(x\right)\\ s_{y}\left(x\right)=\overset{N}{\underset{i=1}{\sum }}\gamma_{i}^{y}\varphi \left(\left\|x-x_{i}\right\|\right)+h_{y}\left(x\right)\\ s_{z}\left(x\right)=\overset{N}{\underset{i=1}{\sum }}\gamma_{i}^{z}\varphi \left(\left\|x-x_{i}\right\|\right)+h_{z}\left(x\right) \end{array}\right.$$

Further details on RBF interpolation as well as applicative solutions can be found in [33].

### 2.2. Kinematics of Large Displacements for a Bi-Dimensional Plate

We suppose that a rectangular plate lies undeformed on the *xy*-plane. A generic transformation moves each point X of the original configuration into point x of the deformed shape. Once displacement field $d\left(X\right)$ is introduced, Equation (4) holds:

(4) $$x=X+d\left(X\right)$$

The plate features only two dimensions, being the thickness very small compared with the in-plane extensions, with the out-of-plane bending being accounted for in the mathematical derivation. If vector $d_{m}={\left\{u\;v\;w\right\}}^{T}$ is the displacement field of the midplane, the deformed coordinate triad can be written in the form:

(5) $$\left\{\begin{array}{c} x\\ y\\ z \end{array}\right\}=\left\{\begin{array}{c} X\\ Y\\ 0 \end{array}\right\}+\left\{\begin{array}{c} u\\ v\\ w \end{array}\right\}+N\cdot Z$$

where N is the unitary vector normal to the deformed mid-plane.

Since large displacements are involved, the Green–Lagrange tensor [34] offers a co-rotational basis for strain evaluation. Deformation gradient F for the points lying on the mid-plane assumes the form:

(6) $$F=\left[\begin{array}{ccc} \left(1+\frac{\partial u}{\partial x}\right) & \frac{\partial u}{\partial y} & \frac{\partial u}{\partial z}\\ \frac{\partial v}{\partial x} & \left(1+\frac{\partial v}{\partial y}\right) & \frac{\partial v}{\partial z}\\ \frac{\partial w}{\partial x} & \frac{\partial w}{\partial y} & \left(1+\frac{\partial w}{\partial z}\right) \end{array}\right]$$

Vector $v_{n}$, normal to the deformed mid-plane, is given by the vector product of the first two columns of F, which map two fibres originally along *x* and *y* into the transformed configuration:

(7) $$v_{n}=\left\{\begin{array}{c} \left(1+\frac{\partial u}{\partial x}\right)\\ \frac{\partial v}{\partial x}\\ \frac{\partial w}{\partial x} \end{array}\right\}\times \left\{\begin{array}{c} \frac{\partial u}{\partial y}\\ \left(1+\frac{\partial v}{\partial y}\right)\\ \frac{\partial w}{\partial y} \end{array}\right\}$$

Hence:

(8) $$N=\frac{v_{n}}{\left\|v_{n}\right\|}$$

Equation (8) enters Equation (5), which represents the transformation of all the points of the plate, including those in the thickness (Z→z). The strain field described holds in the hypothesis of plane deformation ($\varepsilon_{z}=0$); an out-of-plane shrinking can be introduced to consider the hypothesis of plane stress ($\sigma_{z}=0$).

We introduce:

(9) $$G_{1}=\left\{\begin{array}{c} 1\\ 0\\ 0 \end{array}\right\},\;G_{2}=\left\{\begin{array}{c} 0\\ 1\\ 0 \end{array}\right\}\;\mathrm{and}\;G_{3}=\left\{\begin{array}{c} 0\\ 0\\ 1 \end{array}\right\}$$

and:

(10) $$g_{1}=\left\{\begin{array}{c} \frac{\partial x}{\partial X}\\ \frac{\partial y}{\partial X}\\ \frac{\partial z}{\partial X} \end{array}\right\}=G_{1}+\frac{\partial d_{m}}{\partial X}+K_{x}\cdot Z$$

$$g_{2}=\left\{\begin{array}{c} \frac{\partial x}{\partial Y}\\ \frac{\partial y}{\partial Y}\\ \frac{\partial z}{\partial Y} \end{array}\right\}=G_{2}+\frac{\partial d_{m}}{\partial Y}+K_{y}\cdot Z$$

$$g_{3}=\left\{\begin{array}{c} \frac{\partial x}{\partial Z}\\ \frac{\partial y}{\partial Z}\\ \frac{\partial z}{\partial Z} \end{array}\right\}=N$$

where $K_{x}$ and $K_{y}$ collect the terms linear in Z related to bending.

The Green–Lagrange tensor of the transformation in object is:

(11) $$E=\frac{1}{2}\left[\begin{array}{ccc} g_{1}{}^{T}g_{1}-1 & g_{2}{}^{T}g_{1} & g_{1}{}^{T}g_{3}\\ g_{2}{}^{T}g_{1} & g_{2}{}^{T}g_{2}-1 & g_{2}{}^{T}g_{3}\\ g_{3}{}^{T}g_{1} & g_{3}{}^{T}g_{2} & g_{3}{}^{T}g_{3}-1 \end{array}\right]$$

If the curvature radius of bending is large compared with the plate thickness, the hypothesis of small deformations holds, and the terms of second order in Equation (11) can be neglected, with E directly supplying strain quantities.

### 2.3. Analytical Test Cases

In the proposed approach, RBFs are used to represent the large displacement (LD) motion field of a plate body. In this way, all the differential quantities present from Equation (6) onwards are obtained with the analytical differentiation of the radial bases, and no finite differences or other approximations are introduced. This strategy, that approaches LD theory taking advantage of the analytical nature of RBFs, is hereinafter called RBFLD (Radial Basis Function with Large Displacement). The next sections address the analytical and numerical tests carried out to assess the soundness of the RBFLD method. As a first benchmark, two folding transformations, for which both displacements and results were analytically known, were chosen to compare the strain fields obtained using the RBFLD method to those achieved using a commercial FEM solver. In both applications, a thin plate with dimensions a = 0.5 m and b = 1 m and thickness t = 0.005 m was employed, in order to have a thickness–width ratio way lower than 1/20.

#### 2.3.1. Test Case 1

**Figure 2:**
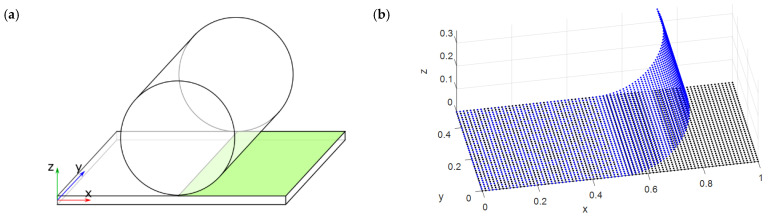
(**a**) Test-case geometry and (**b**) displacement field imposed to RBF centers.

A folding kinematic was first applied to the plate by wrapping it around a cylinder with radius equal to 0.25 m as shown in Figure 2a, where the portion of plate affected by the folding action is highlighted in green.

The RBF displacement field of the plate, modeled by a 40 × 80 grid of points evenly distributed for a total of 3200 centers, was computed by mapping each point on the plane at a certain distance from the cylinder axis to its deformed counterpart along an arc with the same length. The resulting kinematic is shown in Figure 2b, in which the RBF displacement field applied to the source points is shown, with the undeformed plate in black and the final folded position in blue. The motion was applied by taking care not to stretch the middle plane of the plate, simulating a pure rolling action in order to have a straightforward term of comparison of the results with analytical values.

#### 2.3.2. Test Case 2

To bring into play the deformation along the y-direction and a shear deformation along the thickness, another case was studied, by rotating the cylinder around the z-axis by −20°.

**Figure 3:**
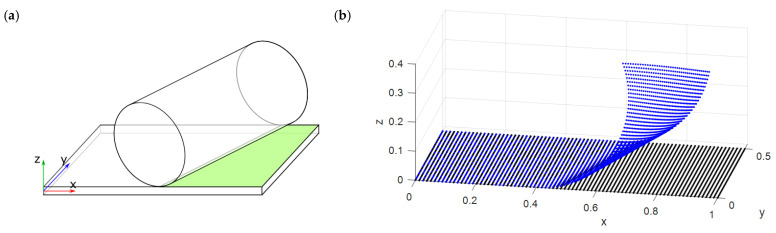
(**a**) Test-case geometry with rotated cylinder and (**b**) displacement field imposed to RBF centers.

In Figure 3, the test-case geometry and its relative RBF displacement field are shown. In addition, in this case, displacements were obtained by computing, for each source point, the shorter distance to the cylinder. For each point, the rotations around the x- and y-axes to be used as inputs to the FEM problem were also collected.

## 3. Results

To perform a comparison with the FEM, a shell model with a number of nodes equal to the introduced RBF centers was created and studied using ANSYS Mechanical APDL, for a total number of 3081 mapped quadrilateral elements modeled using SHELL181 elements. NLGEOM was activated in the solution stage to cope with the large displacements of this problem. For both test cases, the same analytical displacement field used for the RBFLD method was employed for the FEM simulation.

### 3.1. Test Case 1

**Figure 4:**
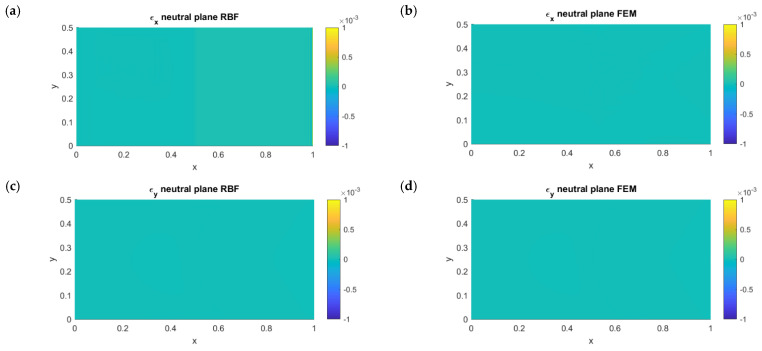
Left: RBF membrane deformations along the x- (**a**) and y-axes (**c**). Right: APDL membrane deformations along the x- (**b**) and y-axes (**d**).

In Figure 4, the results in terms of strain along the x- and y-axes on the neutral plane are shown for both the RBFLD and FEM approaches. As expected, no membrane stretch was present with either method. For a better comparison, the RBFLD and FEM results were plotted with the same contour bounds.

**Figure 5:**
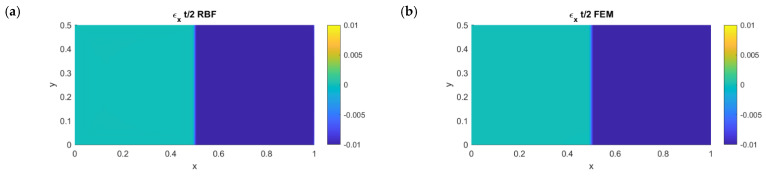
Top strains at t/2 along the x-direction for the RBFLD method (**a**) and the FEM (**b**).

In Figure 5, the $\epsilon_{x}$ results were compared on the top surface for the RBFLD and FEM methods. The constant compression along the x-axis on the area affected by the wrapping motion matched well the analytical value of −0.01, being the result of the rotation of the plate section around the y-axis. In fact, for each angle θ around the cylinder:

(12) $$\epsilon_{x}=\frac{\Delta l}{l}=\frac{t/2\cdot \theta }{r\theta }=\frac{t}{2r}$$

where r is the radius of the cylinder and t the thickness of the plate. Top and bottom strains along the y-direction, as expected, were not present and are not shown for the sake of brevity.

### 3.2. Test Case 2

**Figure 6:**
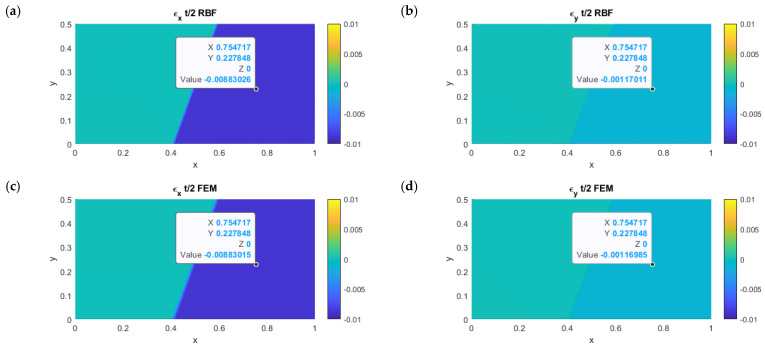
$\epsilon_{x}$ and $\epsilon_{y}$ comparison on the top surface for the RBF and FEM models. From left to right: upper row (**a**,**b**), RBF model; bottom row (**c**,**d**), FEM model.

In order to compare the results for test case 2, in which the cylinder was tilted by an angle θ=20 deg around the z-axis, the strain contours are shown in Figure 6, highlighting an homologous point on the portion affected by the folding. The difference between the two methods was in the order of 0.0012% and 0.0200% for the x- and y-directions, respectively.

Once again, the results were in good agreement with the analytical values; for the top $\epsilon_{x}$ and $\epsilon_{y}$, they were, respectively:

(13) $$\epsilon_{x}=\frac{t}{2r}\cdot \cos^{2}\theta \;\;\mathrm{and}\;\;\epsilon_{y}=\frac{t}{2r}\cdot \sin^{2}\theta$$

This yielded errors of the RBFLD method, with respect to the analytical values, of 0.00043% and 0.02800%. By rotating the cylinder axis, displacements along the x- and y-axes were coupled, and it was possible to observe the generation of a shear deformation on the top and bottom planes.

**Figure 7:**
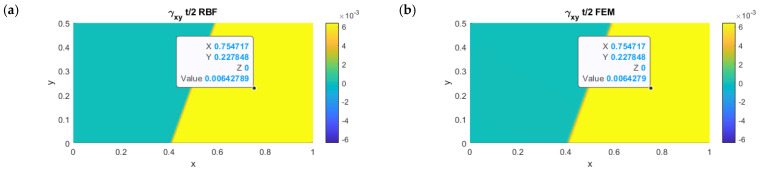
Comparison of in-plane shear deformations for the RBF (**a**) and FEM (**b**) models on the top surface.

In Figure 7a, the comparison between the RBFLD method and the FEM is shown. The results were almost identical to those obtained analytically:

(14) $$\gamma_{xy}=\frac{t}{2r}\cdot \sin\theta \cdot \cos\theta$$

For the RBFLD and FEM methods, the errors were equal to 0.0002% and 0.0004%, respectively.

### 3.3. CAD-Based Test Case

To further demonstrate the method and to test it on a more challenging problem relevant for the flexible-PCB industry, a case in which the plate was bended to a final complex shape known in advance and sketched in the CAD system was considered.

**Figure 8:**
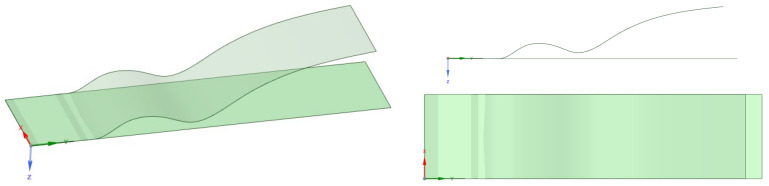
Baseline and target plate geometries.

**Figure 9:**
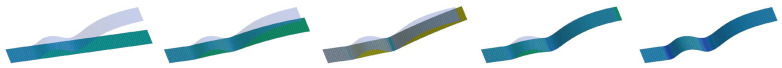
FEM displacement history of the baseline plate.

In Figure 8, the target geometry is shown together with the baseline flat configuration. The final deformed shape was modeled using splines, the longitudinal maintaining of the same length of the undeformed plate being the result of a pure bending motion. This case is representative of a problem in which the plate is deformed according to a predefined kinematic, similarly to a PCB that is closely guided by a mechanical system that does not apply membranal stretch. In order to evaluate the results achieved with the proposed meshless method, a multistep nonlinear FEM analysis was used as a reference. To bring the baseline geometry to the final configuration simulating pure bending, an increasing pressure load was used, maintaining one short edge of the plate fixed and imposing a displacement on the other. The frictionless contact between baseline and target models was assured by generating both meshes with similar spatial discretization. To prevent convergence problems due to instability, the displacement of the free plate end was first only guided along the z-axis—generating a membrane stretch—and then along the y-axis—relieving it. In Figure 9, the deformed-plate shape is shown during the simulation. A perfectly elastic material was taken into account for this application.

To generate the displacement field for the RBFLD method and to have a proper term of comparison, the same spatial discretization of the baseline mesh was used by extracting the nodal positions. A two-step geometrical projection algorithm was then used to calculate a displacement for each node into the final geometry. At first, the nodes on the baseline edges were projected onto the target ones by maintaining the same parametric values. This displacement field was then used to propagate this motion to all the points, obtaining a final distribution very close to the target surface. Then, a final projection step was carried out, pushing all the source points onto the parametric surface along its normal.

**Figure 10:**
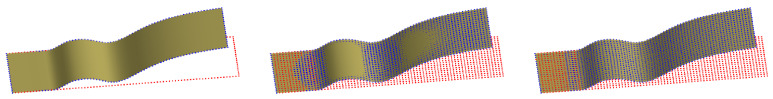
Source points projection from baseline to deformed configuration.

This procedure is illustrated in Figure 10, in which, from left to right, the edge projection, its result on the whole set of points and the result of the final surface projection are shown. The displacements for all the points where then fed to the RBFLD method to retrieve the strain. Being the projection only geometrical and not based on physical behavior, minor membrane stretches were introduced by this process, but being the problem one of pure bending, they were removed from the strain field to restore the expected behavior.

### 3.4. Result

**Figure 11:**
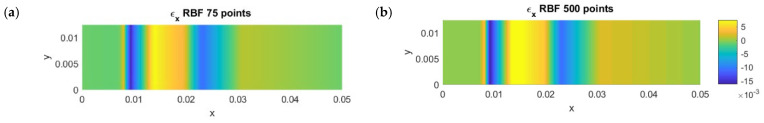

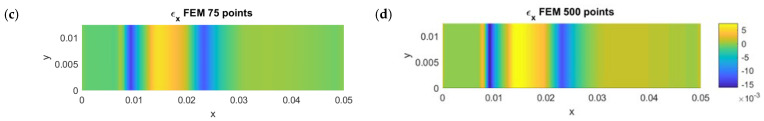
Top strain for the proposed method (**a**,**b**) and FEM (**c**,**d**) for an increasing number of points along the x-direction.

Since the membrane stretches and the top/bottom strains along the y-axis were zero, the results for these fields are not shown. In Figure 11, the contours of the strain along the x-direction are shown for both the RBFLD method and the FEM for two levels of discretization.

For both methods, the results were very similar but were achieved with very different computational costs; while the FEM-based approach requires meshing, problem setup and the solution of a nonlinear problem with contacts, the proposed method can be instead carried out without a structural solver directly in a pre-processing stage, taking advantage on the geometrical definition of CAD. The required time, on the same 10-core machine equipped with an Intel Xeon W-2255 CPU at 3.70 GHz was almost 15 min for the FEM approach and only 25 s for the meshless RBF-based method.

**Figure 12:**
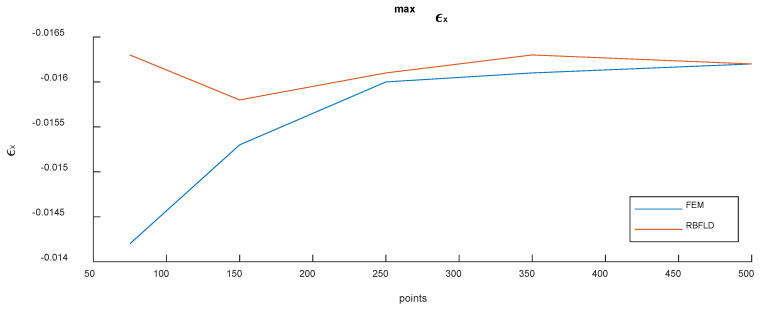
Convergence of the maximum strain for both the FEM and RBFLD methods with the increase in the number of points.

In Figure 12, the convergence behavior of the maximum strain was plotted for both the methods, increasing the number of points of the baseline geometry. Even with a smaller number of points, the RBFLD method was able to catch the maximum strain with good precision, tending to the same value of the FEM with the increase in the level of discretization. This result is in accordance with those achieved in [26], in which it is demonstrated that RBFs converge faster than the FEM for stresses and strains.

## 4. Conclusions

In this paper, a method for the fast computation of strains due to large rotation of thin plates was designed. The discussed strategy, named RBFLD method, relied on RBF interpolation, having its strength in the meshless nature of the representation. A comparison against the FEM was conducted, at first, for two simple cases for which an analytical solution in terms of displacements and strains was available. Then, a more challenging problem relevant for the flexible-PCB industry, in which a known original and deformed CAD geometry was known, was analyzed. In both cases, the results between the RBFLD method and the FEM were comparable but at very different computational costs; with the machine being equal, the RBFLD method took 25 s, while 15 min were required for a nonlinear contact FEM simulation. For the CAD-based RBFLD workflow, the strain field could be computed directly from the geometries, not relying on a numerical grid, while when using the FEM, there was the need for solving a multistep analysis with contacts. As already stated in the pertinent literature, RBF computation demonstrated faster convergence than the FEM.
